# New Insight to the Effects of Heat Treatment in Air on the Permeation Properties of Thin Pd77%Ag23% Membranes

**DOI:** 10.3390/membranes8040092

**Published:** 2018-10-10

**Authors:** Nicla Vicinanza, Ingeborg-Helene Svenum, Thijs Peters, Rune Bredesen, Hilde Venvik

**Affiliations:** 1 Department of Chemical Engineering, Norwegian University of Science and Technology, 7491 Trondheim, Norway; niclavicinanza@gmail.com (N.V.); Ingeborg-Helene.Svenum@sintef.no (I.-H.S.); 2 SINTEF Industry, P.O. Box 124 Blindern, N-0314 Oslo, Norway; Thijs.Peters@sintef.no (T.P.); Rune.Bredesen@sintef.no (R.B.)

**Keywords:** Pd-Ag membranes, hydrogen permeation, surface characterization, solubility, heat treatment

## Abstract

Sputtered Pd77%Ag23% membranes of thickness 2.2–8.5 µm were subjected to a three-step heat treatment in air (HTA) to investigate the relation between thickness and the reported beneficial effects of HTA on hydrogen transport. The permeability experiments were complimented by volumetric hydrogen sorption measurements and atomic force microscopy (AFM) imaging in order to relate the observed effects to changes in hydrogen solubility and/or structure. The results show that the HTA—essentially an oxidation-reduction cycle—mainly affects the thinner membranes, with the hydrogen flux increasing stepwise upon HTA of each membrane side. The hydrogen solubility is found to remain constant upon HTA, and the change must therefore be attributed to improved transport kinetics. The HTA procedure appears to shift the transition from the surface to bulk-limited transport to lower thickness, roughly from ~5 to ≤2.2 µm under the conditions applied here. Although the surface topography results indicate that HTA influences the surface roughness and increases the effective membrane surface area, this cannot be the sole explanation for the observed hydrogen flux increase. This is because considerable surface roughening occurs during hydrogen permeation (no HTA) as well, but not accompanied by the same hydrogen flux enhancement. The latter effect is particularly pronounced for thinner membranes, implying that the structural changes may be dependent on the magnitude of the hydrogen flux.

## 1. Introduction

Palladium-based membranes have been the focus of many studies due to their high hydrogen permeability and selectivity, which may find application in efficient separation technologies [1,2]. At temperature below ~300 °C and pressure below ~2 MPa, however, pure palladium undergoes the α-to-β phase transition that results in irreversible lattice strain. Over time, cycling of the temperature causes the Pd to become brittle; leading to fractures. In order to prevent hydrogen embrittlement, Pd is conveniently alloyed with other metals [3,4]. Silver is a widely used alloying element, reducing the α-to-β phase transition to below room temperature. Moreover, Pd-Ag alloys exhibit higher hydrogen permeability than pure palladium [4,5,6], with a maximum at ~23 wt.% of Ag [6].

Hydrogen flux can be also enhanced by heat treatment in air (HTA) procedures [7,8,9,10,11,12,13,14,15,16,17,18,19], which are essentially oxidation-reduction cycles. For thin membranes, typically in the range of 1–3 µm, the total hydrogen flux through the membrane can be doubled upon air thermal treatment [8,9,10,11,16], and HTA has also been used to regenerate deactivated membranes [14,20,21]. Moreover, Mejdell et al. reported a significant reduction of the CO inhibitive effects on hydrogen permeation through a ~3 µm Pd-Ag (23%) membrane after HTA [9]. The phenomena behind these advantageous effects are, however, still not clear. Different hypotheses have been suggested to explain the influence of heat treatment in air; including cleaning of the Pd-Ag surface, microstructural rearrangement, and induced segregation of Pd towards the surface. Oxidation is known to remove certain impurities and poisoning species from Pd-based surfaces [5,15,16,20]. This can facilitate and improve hydrogen flux, but is not a sufficient explanation that can fully elucidate the positive effects of HTA [14].

Microstructural changes are commonly observed in Pd-based membranes after air exposure, while the permeation properties are maintained or enhanced and even the stability/durability seems upheld. These include increase in surface roughness [10,13,14,17,22,23], as well as defect/void formation [8,11,14,17,19]. The increased surface roughness leads to an enlarged active surface area which can promote the hydrogen flux through the membrane given that surface phenomena are transport limiting. Furthermore, surface roughening is associated with grain growth in the membrane bulk [10,13,14,17,19]. As there is no clear agreement on whether larger grains inhibit [24,25,26] or enhance [27,28] hydrogen flux, it is not possible to irrefutably attribute hydrogen flux enhancement to formation of larger grains. Recently we established, however, a possible correlation between the solubility of hydrogen and the average grain boundary density in sputtered membranes, rendering the diffusivity practically unaffected as long as the hydrogen transport was controlled by bulk diffusion [29]. On the other hand, Zhang and co-workers [17] reported an increase in the hydrogen sorption kinetics that was attributed to higher hydrogen diffusivity for a 25 µm cold-rolled Pd-Ag 25 wt.% membrane after heat treatment in air at 300 °C for 1 day.

Another hypothesis is related to surface segregation of Pd as a result of the heat treatment in air, since H_2_ dissociates over and binds to Pd but not to Ag. It is well established that Ag segregates to the membrane surface of ideal Pd-Ag alloys in absence of adsorbates; i.e., under vacuum or inert gas conditions, while a reverse segregation of Pd is induced after exposure of Pd-Ag surfaces to hydrogen and several other chemisorbing species [30,31,32]. The thermal treatment in air has been found to yield formation of a ~2 nm thick PdO layer [13] on the Pd-Ag surface and to an enrichment of Pd that can be involved in the enhancement of hydrogen flux through Pd-Ag membranes [13,15,16,32]. Segregation and rearrangement of the outmost atomic layers could also be linked, in the sense that this could affect in particular the transfer of hydrogen from the surface to the bulk or vice versa; processes that are particularly difficult to study experimentally.

In this work, a new approach to the heat treatment in air procedure has been applied in order to further investigate its effect on hydrogen transport properties in Pd-Ag membranes. The procedure was performed so that each side of the membranes was consecutively exposed to ambient air in order to probe the individual surface responses, which to our knowledge have not been previously addressed in the literature. A final HTA applied to both sides together was also performed in order to establish if additional effects exist. Different membrane thicknesses have been included to the investigation, as well as measurements of hydrogen solubility before and after heat treatment, to better understand the transport mechanisms involved. The membranes were characterized using atomic force microscopy (AFM) for as-grown membranes, hydrogen-stabilized, and heat-treated in air (with subsequent hydrogen stabilization), to monitor changes in surface topography.

## 2. Materials and Methods

### 2.1. Membrane Preparation

Pd77%Ag23% thin, self-supported membranes were produced by SINTEF using a unique two-step magneton sputtering technique onto silicon single crystal substrates [33,34]. Pd-Ag films with thicknesses of 2.2, 4.7, 6.9, 8.5, and 11.2 µm were studied. The membrane thickness was determined using white light interferometry. In this work, the membranes analyzed have been categorized as follows: ‘as-grown’ refers to Pd77%Ag23% thin film samples just pulled-off from the silicon substrate; ‘hydrogen-stabilized’ refers to membranes exposed to hydrogen permeation measurements only; ‘air-treated’ corresponds to membranes that have been heat-treated in air (HTA) and subsequently stabilized under hydrogen according to the procedures described below. The ‘growth/feed side’ of the membranes is the side growing during the magneton sputtering deposition and was always exposed to the feed gas during permeation experiments. The ‘substrate/permeate side’ refers to the membrane side facing the silicon wafer under fabrication, and was always kept to the permeate (low pressure) side of the membranes during permeation experiments.

### 2.2. Hydrogen Stabilization/Permeation

**Figure 1:**
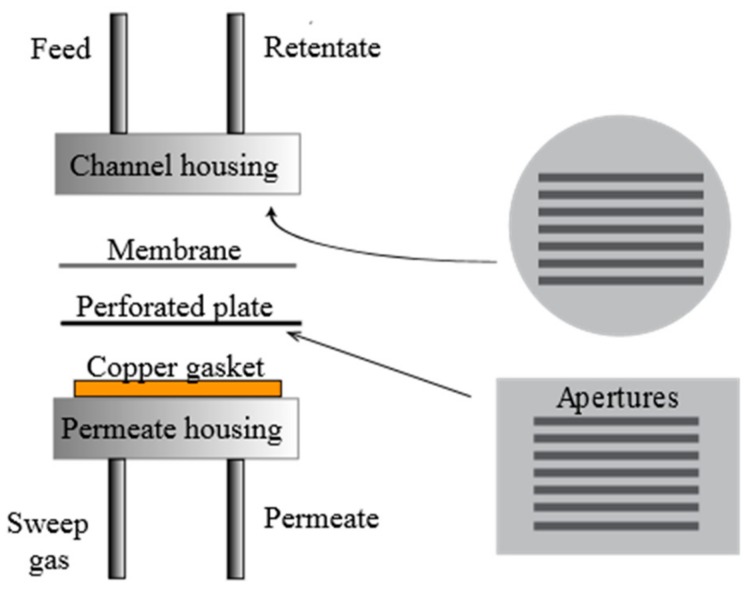
Sketch of the microchannel reactor configuration.

The permeation behavior under pure hydrogen (purity 99.999%) through the Pd77%Ag23% membranes was studied as a function of temperature and pressure with no use of sweep gas. All membranes were mounted in a microchannel configuration as depicted in Figure 1. The membranes were placed in between a polished stainless steel feed housing and a polished stainless steel plate. The feed housing had seven channels for gas flow corresponding to a total active surface area of 0.91 cm^2^, in accordance with the permeate side steel plate geometry. On the permeate side, an open stainless steel housing was sealed to the perforated steel plate by a polished copper gasket. In conjunction with absence of sweep gas or dilutants, this configuration enables investigation of very thin membranes in absence of transport limitations from the gas phase (concentration polarization) or the support [35]. Membrane leakage was checked by using an Agilent 490 Micro-GC (Agilent Technologies, Santa Clara, CA, USA). No leakage could be detected during any of the permeation experiments both before and after the three-step heat treatment procedure. Experiments were performed at selected temperatures of 300, 350, and 400 °C. 300 °C was reached by ramping at 2 °C per minute with nitrogen (purity 99.999%) flushing on the feed side and argon (purity 99.999%) on the permeate side. After reaching 300 °C, nitrogen and argon were slowly removed from the system and hydrogen introduced. The permeate side was left at atmospheric pressure while a differential pressure of maximum 2 bar was applied on the retentate side. The permeate flow was measured by a bubble flow meter.

### 2.3. Heat Treatment in Air (HTA)

The three-step heat treatment in air (HTA) procedure was performed between hydrogen permeation experiments for three selected membrane thicknesses; 2.2, 4.7, and 8.5 µm respectively. The HTA was mainly performed using the following sequence: (i) permeate side, (ii) feed side, (iii) both sides one more time, with hydrogen permeation experiments between each step. Each HTA step was carried out at 300 °C for one hour. Before exposing the membranes to ambient air, hydrogen was removed from the system by introducing nitrogen on the feed side and argon on the permeate side for about 15 min. Nitrogen/argon were reintroduced for 15 min at the feed/permeate sides in order to flush out air. Hydrogen was then introduced again and nitrogen/argon slowly removed. As will be shown, HTA had the largest effect for the thinnest membranes. Another 2.2 µm membrane was therefore subjected to an alternative HTA sequence, denoted HTA2: (i) feed side, (ii) permeate side, (iii) both sides one more time, again with hydrogen permeation experiments between each step and procedures otherwise as described above.

### 2.4. Hydrogen Solubility

Equilibrium sorption measurements were carried out as described in [29] using an ASAP 2020 chemisorption analyzer (Micromeritics Instrument Corporation, Norcross, GA, USA) for 2.2 µm and 8.5 µm as-grown membranes, as well as after heat treatment in air. The heat treatment in air for the sorption samples was performed in a furnace at 300 °C under ambient air for one hour. Volumetric sorption was performed with a hydrogen pressure between 0.02 and 90.7 kPa. In every measurement, a sample mass close to 0.1 grams was used, taking into account also mass loss from degassing of the sample. The sorption measurements were carried out twice at three different temperatures: 300, 350, and 400 °C.

### 2.5. AFM Imaging

The surface topography was investigated by atomic force microscopy (AFM, Bruker, Boston, MA, USA) using a Multimode AFM instrument with a Veeco Multimode controller. All force spectroscopy analysis was performed in tapping mode under atmospheric conditions. Surface topography was investigated for both growth/feed side and substrate/permeate side for all the as-grown membranes, for hydrogen stabilized membranes and selected membranes after heat treatment in air with subsequent hydrogen stabilization. At least five surface scans were obtained at different locations for each sample. The first flattening order, provided by the AFM-instrument software (Nanoscope Software Version 7.2, by Veeco, Plainview, NY, USA), was performed in order to remove tilt and noise from all images. Surface roughness was estimated as the root mean square roughness (Rq) from the measured AFM images.

## 3. Results and Discussion

### 3.1. Permeability

Hydrogen permeation through Pd-Ag membranes generally follows the solution-diffusion mechanism, where Fick’s law of diffusion describes the mass transport

(1) $$J_{H_{2}}=\frac{P}{t}\left(p_{1}^{n}-p_{2}^{n}\right)=\frac{SD}{t}\left(p_{1}^{n}-p_{2}^{n}\right)$$

*P* is the permeability of the membrane, *t* the thickness, *p*_1_ and *p*_2_ the partial pressure of hydrogen on the high pressure side and low pressure side of the membrane, respectively, *S* the solubility, and *D* the diffusion coefficient. The *n*-value is determined by the rate-limiting step of the transport mechanism as explain further below. The diffusivity can be expressed as

(2) $$D=D_{0}\exp\left(-\frac{E_{a}}{RT}\right)$$

where *D*_0_ is a pre-exponential factor and *E_a_* the activation energy for diffusion.

**Figure 2:**
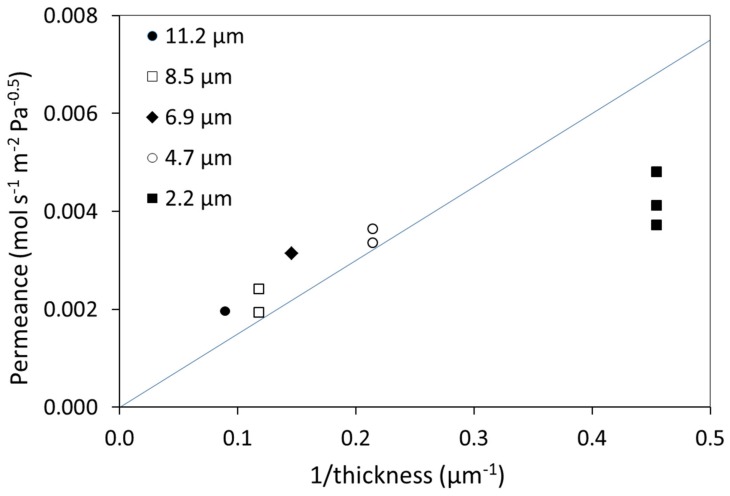
Measured hydrogen permeance as function of inverse thickness at 300 °C for membranes not subjected to HTA. The full line refers to values of permeance reported if bulk is the rate limiting step (1.5 × 10^−8^·mol·s^−1^·m^−2^·Pa^−0.5^) [5].

Hydrogen permeance *(P*/*t*, Equation (1)) values, measured at 300 °C for untreated membranes (not subjected to HTA)*,* are plotted in Figure 2 as a function of inverse thickness. In the calculation of the permeance, *n* = 0.5 was applied, which is essentially valid only with bulk diffusion as the rate-limiting step [36,37,38,39,40,41]. When the kinetics is bulk-limited, the permeance should be proportional to the inverse thickness, leading to a constant value for the permeability (a material property). In thick Pd-membranes, hydrogen generally forms a dilute solution and the transport is bulk-limited. The full line (Figure 2) refers to a bulk value of permeability equal to 1.5 × 10^−8^ mol·m·m^−2^·s^−1^·Pa^−0.5^ that has been reported for a 100 µm thick Pd77%Ag23% membrane at 300 °C [5]. A previous investigation of pure Pd membranes in the thickness range 10 to 150 µm indicated that bulk diffusion was rate limiting for thicknesses above 20 µm [37]. As the thickness decreases, the transport mechanism may be controlled by a combination of surface effects and bulk diffusion (0.5 < *n* < 1) [42,43]. Eventually, surface phenomena such as adsorption/dissociation and/or association/desorption become completely rate-limiting, and the pressure exponent approaches unity [36,37,38,39,40,41]. Figure 2 indicates a bulk limited transport with permeance values somewhat higher than the bulk literature value for the thicker membranes (≤4.7 µm), while the thinnest membranes exhibit permeance values indicative of surface limitations affecting hydrogen permeation. The thickness at which this transition appears may depend both on the conditions (T, P, H_2_ feed content) and the material properties as affected by the fabrication and eventual pre-treatment of the membrane. Several studies report that surface phenomena start to have an impact from 4–5 µm [10,29,40,44].

There are some variations in the measured permeance between membranes of the same thickness; larger for the 2.2 µm membrane relative to the 4.7 µm and 8.5 µm membrane. There could be several reasons for this variation, including experimental uncertainty. There may be a gradient in membrane thickness as large as ~10% across the silicon wafer, but we take care to mainly utilize the mid-sections for permeation experiments to reduce this deviation. Time and contamination during ambient storage may have an effect, as well as slight stretching of the material under total pressure difference [10]. Such sputtered membranes of similar thickness are, however, not found to exhibit major differences in microstructure, composition, or surface topography [10,45,46], but a general observation that will be further discussed below is that this variation between principally equivalent samples is also reduced by HTA [10].

**Figure 3:**
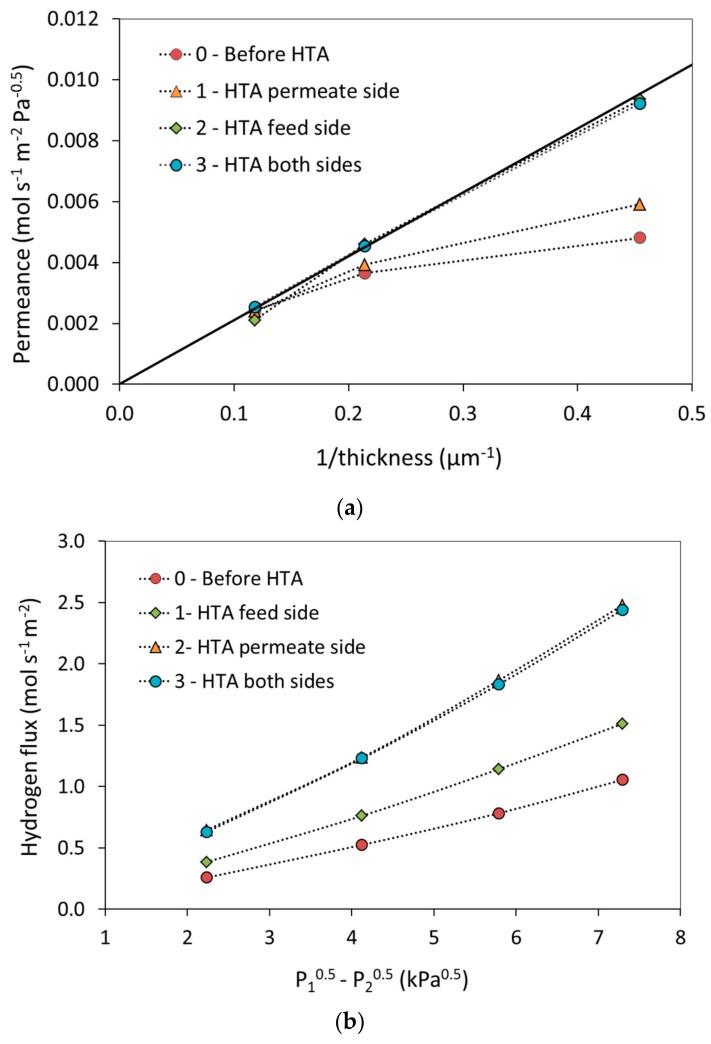
Permeance measured at 300 °C for each single step of heat treatment in air; (**a**) as function of inverse thickness for the main HTA membrane side sequence with the full line indicating a permeability of 2.1 × 10^−8^ mol·s^−1^·m^−2^·Pa^−0.5^, and (**b**) as function of the difference in the square root of the hydrogen partial pressure for the HTA2 membrane side sequence applied to a 2.2 µm thick membrane.

After stabilization under hydrogen and measurement of the permeance, membranes with thicknesses of 2.2, 4.7, and 8.5 µm were subjected to the three-step HTA procedures as described above. The results are displayed in Figure 3 and Table 1. Figure 3a shows the hydrogen permeance plotted as a function of inverse thickness before HTA as well as after each step in the HTA procedure; *n* assumed equal to 0.5 also here. HTA has larger effect as the thickness decreases, with practically no effect for the 8.5 µm thick membrane. Moreover, the permeance increases after each of the first two HTA steps, i.e., upon oxidation-reduction of each side of the membrane. The first increase amounts to ~20% (HTA permeate/substrate) while the second (HTA feed/growth) is 60–40% (Table 1), depending on temperature, for the 2.2 µm membrane. There is no significant change when both sides are simultaneously exposed one more time to air. Figure 3a also implies that the permeance is inversely proportional to the membrane thickness after HTA. Hence, the oxidation-reduction cycle imposed by the HTA procedure apparently shifts the surface transport limitation to lower thickness and bulk diffusion becomes rate-limiting for the hydrogen transport over the whole thickness range and experimental conditions investigated. Moreover, the surface limitations appear to impose transport limitations on both sides initially, which can be lifted stepwise by oxidizing one side at the time.

In order to investigate the importance of the order of the heat treatment with respect to growth/feed or substrate/permeate side of the membrane an experiment in the opposite order (HTA2) was performed for the 2.2 µm thickness as mentioned above. The results are displayed in Figure 3b and in Table 1. The stepwise increase in permeability is definitely maintained, possibly with some differences due to the order, i.e., feed or permeate side first. The relative increases are now ~40% (HTA feed/growth) followed by 70–30% (HTA permeate/substrate) with increasing temperature, to eventually reach similar permeances values as for the main HTA sequence. Conclusions with respect to order are, however, complicated by the variation in values obtained for the hydrogen stabilization as discussed above.

The dissociative adsorption of hydrogen over palladium surface atoms is practically non-activated [47]. It is thus difficult to ascribe the observed permeation increase on the feed side for either of the sequences in terms of adsorption properties only. Moreover, the hydrogen-stabilization as well as the HTA should have promoted Pd termination of the surface over Ag, but (temperature dependent) coverage effects may complicate the segregation behavior [48]. Nevertheless, the subsurface structure and elemental distribution may also be affected by the treatment and play a role in the transfer from the surface to the bulk and may affect both sides.

After exposure to air of both sides of the membranes, the permeability approaches a value of 2.1 ± 0.1 × 10^−8^·mol·s^−1^·m^−2^·Pa^−0.5^ at 300 °C, as shown by Table 1 as well as the values for the 4.7 µm thickness (not shown). This is comparable to previous values reported in literature for similar membranes [10], for which the HTA also seems to partially diminish the variations between samples from different wafers or fabrication batches that is observed during the initial stabilization under hydrogen. The oxidation-reduction cycle may hence induce a higher degree of structural and compositional uniformity. The flux increase after HTA of both sides of the thinnest membrane corresponds to a doubling of the permeability, also in accordance with what has been previously reported in literature [10,13,14,16], although a comparison of permeabilities based on *n* equal to 0.5 is not valid in the strictest sense. The final permeability value at 300 °C is, however, higher than the value of 1.5 × 10^−8^·mol·m·m^−2^·s^−1^·Pa^−0.5^ at 300 °C expected for bulk-limited transport reported in literature [5]. This could be related to differences in grain structure/density [29], grain orientation (the sputtered PdAg films contain predominantly grains oriented along <111> parallel/normal to the surface [49]), as well as purity, but requires further investigation.

### 3.2. Hydrogen Solubility and Diffusivity

In addition to hydrogen permeation experiments before and after the three-step heat treatment procedure, hydrogen sorption measurements were carried out to investigate possible variation caused by the HTA. The hydrogen solubility values were obtained before and after heat treatment in air of both sides simultaneously for 2.2 µm and 8.5 µm membranes, and the Sieverts’ constants are reported in Table 2.

Solubility decreases with increasing temperature, as expected [3,50,51,52,53,54]. Moreover, the solubility increases as the thickness decreases as we have recently reported [29]. These thickness dependent changes in solubility were related to differences in the grain structure of the sputtered Pd-Ag films. After HTA of both sides, the values of Sieverts’ constant remain basically unaffected for 300 and 400 °C. There is, however, some increase for the values at 350 °C, especially for the 8.5 µm. We are unsure whether this discrepancy can be considered an experimental error, but the data are not sufficient to conclude on significant HTA-induced changes in solubility. Previous results for a 25 µm cold-worked Pd-Ag25 wt.% indicated no change in hydrogen solubility after heat treatment in air [17,18].

**Figure 4:**
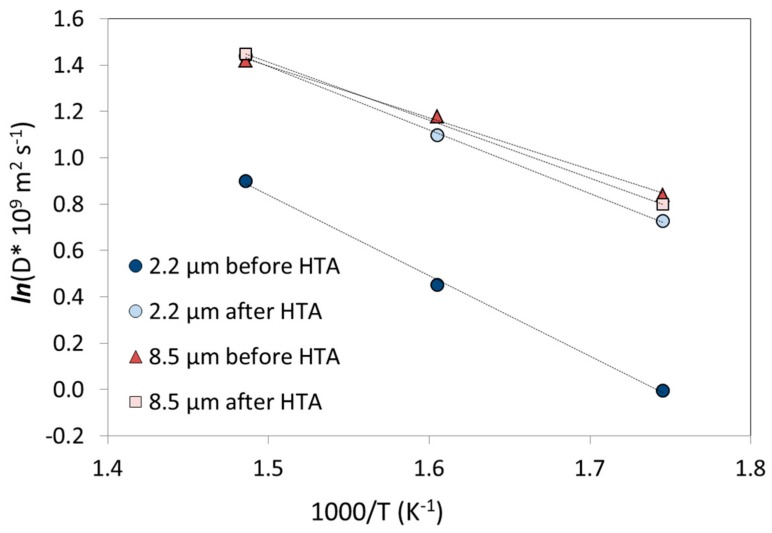
Arrhenius plot of the diffusivity for 2.2 µm and 8.5 µm thick membranes before and after HTA of both sides for the temperature range 300–400 °C. The diffusivity scale is presented in logarithmic form and the dotted lines are linear fits.

Hydrogen diffusivities calculated based on reaction (1) for the 2.2 µm and 8.5 µm thick membranes before and after HTA are shown in Figure 4 as a function of inverse temperature. Estimated values for the pre-exponential factor (*D*_0_) and activation energy (*E_a_*) using Equation (2) are listed in Table 3. There are no significant changes found in diffusivity for the 8.5 µm membrane as both the permeability (Table 2) and solubility (Table 3) are constant before and after HTA. The solubility value obtained after HTA at 350 °C has been omitted from the fit in Figure 4, but the values remain if it is taken in. The apparent activation energy of 20 kJ/mol (before and after HTA) is comparable to results obtained for Pd-Ag with bulk-limited kinetics. Völkl and Alefeld collected data for the diffusion coefficient of hydrogen in Pd from 25 authors and estimated a mean value of *E_a_* ~ 22.4 kJ/mol and *D*_0_ ~ 2.9 × 10^−7^ m^2^/s [55]. Moreover, Holleck calculated for a Pd80%Ag20% (0.08–0.20 cm thick) membrane values of *E_a_* of ~22.3 ± 0.4 kJ/mol and *D*_0_ ~ (2.33 ± 0.2) × 10^−7^ m^2^/s [56]. This suggests that HTA does not change the hydrogen transport mechanism for thick sputtered Pd77%Ag23% membranes.

In the case of the 2.2 µm thick membrane, the permeability enhancement must be attributed to an apparent increase in the diffusivity since the solubility seems to remain constant. Both the pre-exponential factor and the activation energy changes after HTA: *E_a_* decreases from 29 kJ/mol before HTA to 24 kJ/mol after HTA on both sides, and similar values were obtained for the opposite order of stepwise air-treatment (HTA2). Since *E_a_* exceeds the values reported for thicker membranes in literature [56], this supports the idea that surface phenomena are rate-limiting before HTA. However, if the enhanced kinetics of hydrogen transport upon heat treatment in air is associated with a transition from surface to bulk limited transport, there should be a change in the *n*-value that essentially complicates the comparison by fitting. However, comprehensive analysis to extract *n*-values also requires a larger pressure range that what could been applied here [11,35]. Nevertheless, the activation energies associated with (associative) desorption should be generally higher than those for diffusion, but may depend on the surface composition (Pd/Ag) as well as the coverage as discussed in [29].

### 3.3. Surface Topography

**Figure 5:**
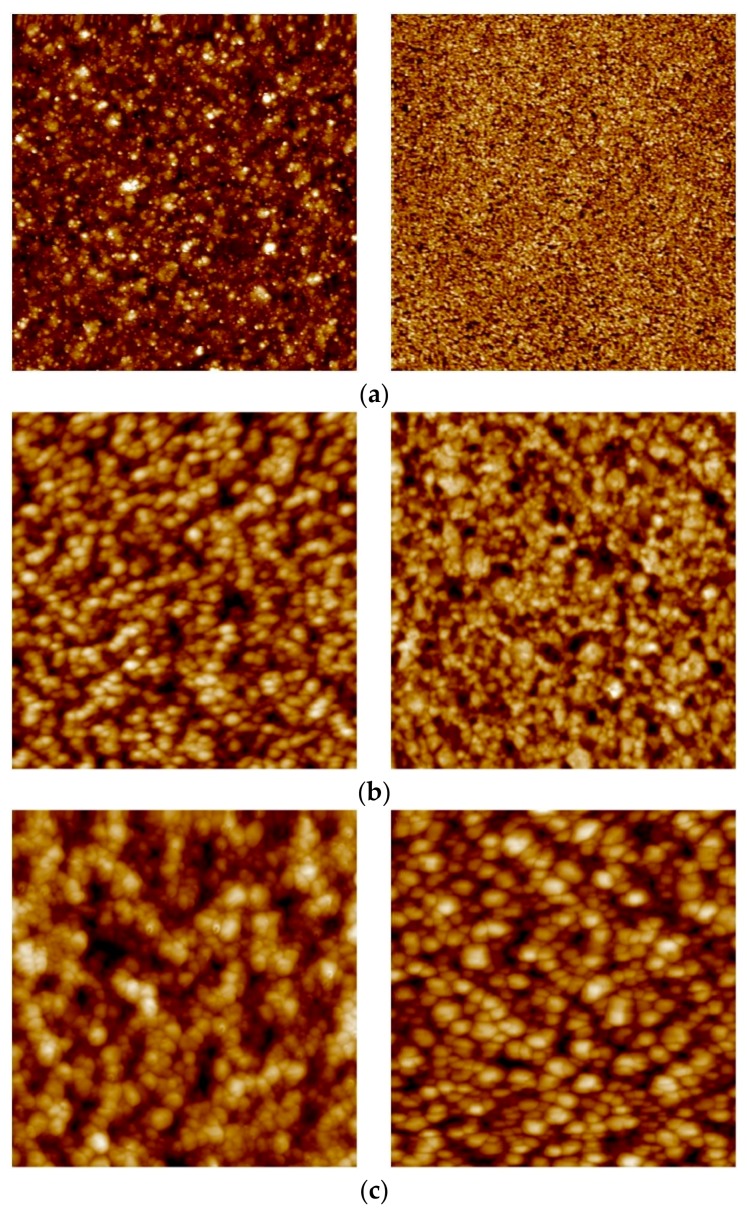
AFM images of the 2.2 µm thick membrane for the feed/growth side (left panel) and permeate/substrate side (right panel) for (**a**) as-grown; (**b**) hydrogen stabilized; and (**c**) HTA-subjected samples. Image areas: (**a**) right: 1 × 1 µm^2^; rest: 5 × 5 µm^2^.

**Figure 6:**
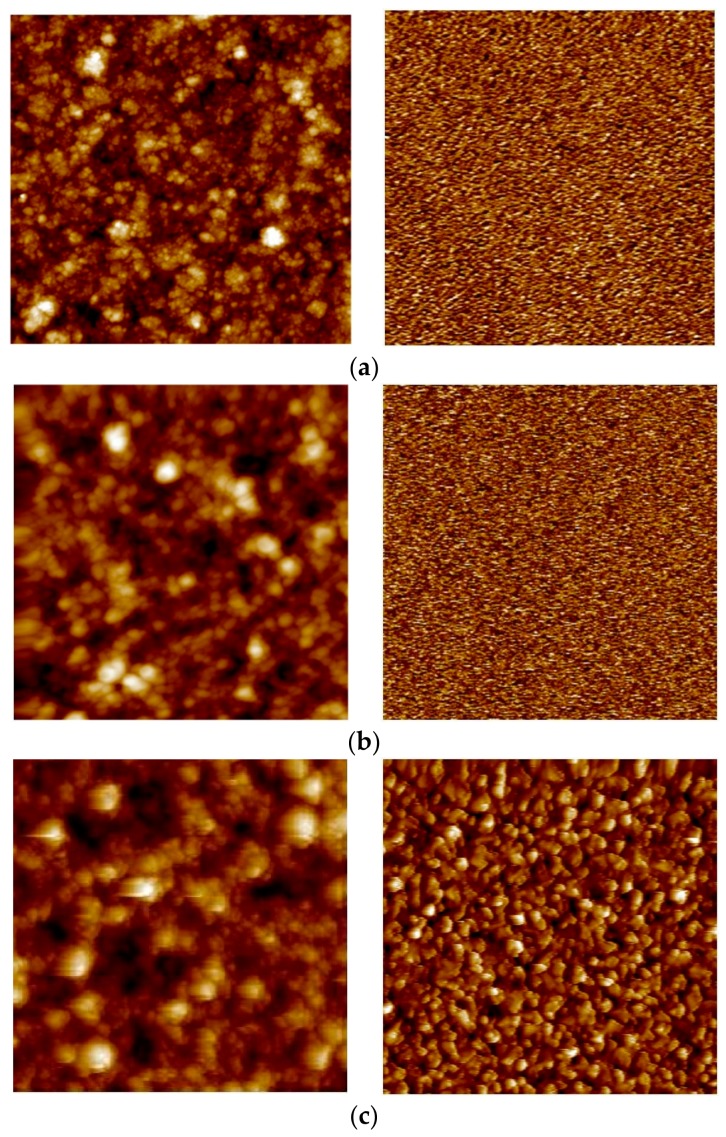
AFM images of the 8.5 µm thick membrane for the feed/growth side (left panel) and permeated/substrate side (right panel) for (**a**) as-grown; (**b**) hydrogen stabilized; and (**c**) HTA-subjected samples. Image areas: (**a**) right and (**b**) right: 1 × 1 µm^2^; rest: 5 × 5 µm^2^.

AFM was used in order analyze how the hydrogen stabilization and HTA procedure affects the surface topography of the different membranes. Figure 5 and Figure 6 show representative AFM images of 2.2 µm and 8.5 µm thick as-grown, hydrogen-stabilized, and air-treated membranes. The corresponding roughness values of all the measured membranes are reported in Table 4. The feed/growth side of as-grown samples shows an increase in the surface roughness as the thickness increases. The corresponding permeate/substrate side is very smooth (0.2–0.4 nm) with some variation in surface roughness between the different samples and thicknesses analyzed. This is in accordance with previous results for similar, sputtered membranes [10,29], and reflects the nature of the nucleation and growth phenomena involved during sputtering. A relatively high density of nuclei seem to form on the substrate, and then growth proceeds by continuing some grains while others are terminated [19].

Upon hydrogen permeation, the feed side surface roughness is found to increase moderately, while the effect of H_2_ stabilization on the permeate side is found to be strongly thickness dependent. Hydrogen exposure/permeation has already been reported to increase the surface roughness of 1.3 µm sputtered Pd-Ag films [10]. For thick membranes (≥8.5 µm), hydrogen exposure causes only small changes to the surface structure. The permeate side becomes more roughened as the thickness decreases and the surface roughness of the 2.2 µm membrane becomes comparable for the two opposite sides. The reason behind this trend is not known, and it has—to our knowledge—not been reported before. The thickness dependent surface roughening on the permeate side under hydrogen permeation may possibly be connected to the fact that thinner membranes experience higher hydrogen flux than thicker membranes. In general, substantial roughening is associated with grain growth [19,29] and the higher hydrogen flux should hence facilitate restructuring and grain growth.

After the HTA procedure and subsequent hydrogen stabilization the surface topology undergoes further changes, depending on thickness. For the thickest (8.5 µm) membrane analyzed, the roughness of the feed side is mainly enhanced upon exposure to hydrogen while the HTA procedure has only minor effect (Figure 5). The permeate side, on the other hand, is strongly roughened after HTA. The feed side of the 4.7 µm thick membrane, already roughened during the hydrogen stabilization step, has yet another increase in roughness once oxidized. A similar behavior is also observed for the permeate side. Heat treatment in air contributes to a strong additional increase in surface roughness of feed side for the 2.2 µm membrane (Figure 4, Table 2). The permeate side roughness, instead, is enhanced only after hydrogen exposure to around 12 nm, and no further increase after HTA is observed.

Eventually, upon HTA the roughness values end up in the range of 20–30 nm on the growth/feed side and 9–15 nm on the substrate/permeate side, irrespective of the thickness dependent differences existing due to the growth process or developing during H_2_ stabilization only. This is in agreement with several other investigations [10,13,14,17,22,23], reporting that the heat treatment in air helps the overall surface roughening process, thus creating new active sites and an increase of surface area. However, the roughening occurs also during hydrogen permeation—in particular for the thinner membranes—without observing an associated strong increase in flux. The permeation enhancement effect of the heat treatment in air can therefore not be fully attributed to an increase in surface area [14,19,57].

## 4. Conclusions

A three-step heat treatment in air has been performed on sputtered Pd77%Ag23% membranes with thickness ranging from 2.2 µm to 8.5 µm. The HTA is found to increase hydrogen permeability after each of the steps and to have a larger effect as the membrane thickness decreases. Air oxidation has no apparent effect on the hydrogen solubility, implying that enhancement in permeability may be related to an increase in diffusivity for thinner membranes (≤5 µm). The data also suggest that bulk diffusion is the rate-limiting step for transport after HTA for all membranes, while surface phenomena are rate-limiting for thinner membranes prior thermal air treatment. Moreover, AFM studies reveal that the HTA increases the surface roughness of the membranes. However, significant surface roughening is already experienced upon stabilization under hydrogen, in particular for the thinner membranes, indicating that increase in permeability is not only attributable to increased surface area.

## Figures and Tables

**Table 1:** Permeability measured at 300 °C after before, between and after each of the three steps in the main HTA membrane side sequences for the 2.2 µm thick membranes.

T (°C)	Permeability 10^8^ (mol·m·m^−2^·s^−1^·Pa^−0.5^)							
	Main HTA Sequence				HTA2 Sequence			
	Before	Feed	Perm	Both	Before	Feed	Perm	Both
300	1.1	1.3	2.1	2.0	0.9	1.3	2.2	2.1
350	1.2	1.5	2.3	2.3	1.1	1.6	2.4	2.3
400	1.5	1.8	2.5	2.5	1.5	2.1	2.7	2.7

**Table 2:** Sieverts’ constant measured at different temperature for 2.2 µm and 8.5 µm thick membranes before and after heat treatment in air.

Sample Thickness (µm)	Temperature (°C)	Sieverts’ Constant (µmol/g·Pa^0.5^)	
		Before HTA	After HTA
8.5	300	0.77	0.79
	350	0.53	0.63
	400	0.44	0.45
2.2	300	0.82	0.81
	350	0.57	0.60
	400	0.47	0.47

**Table 3:** Estimated values of the pre-exponential factor (*D*_0_) and of the activation energy (*E_a_*) for 2.2 µm and 8.5 µm thick membranes before and after the main HTA sequence.

Sample Thickness (µm)	Before HTA		After HTA	
	*D*_0_ (m^2^/s)	*E_a_* (kJ/mol)	*D*_0_ (m^2^/s)	*E_a_* (kJ/mol)
2.2	4.9 × 10^−7^	29	3.6 × 10^−7^	24
8.5	1.5 × 10^−7^	20	1.7 × 10^−7^	20

**Table 4:** Surface roughness for as-grown membranes, after hydrogen stabilization and HTA with subsequent hydrogen stabilization obtained from AFM imaging analysis for both the growth/feed and substrate/permeate side. The analyzed areas are based on (5 × 5) µm^2^ images except for the substrate/permeate side as-grown membranes where images with an area of (1 × 1) µm^2^ are used.

Membrane Thickness (µm)	Roughness (nm)					
	As-Grown		Hydrogen Stabilization		HTA	
	Growth/Feed	Substrate/Permeate	Growth/Feed	Substrate/Permeate	Growth/Feed	Substrate/Permeate
2.2	8.4 ± 0.3	0.29 ± 0.02	13.8 ± 0.8	12.3 ± 0.6	20.3 ± 1.3	11.6 ± 0.6
4.7	10.7 ± 0.6	0.19 ± 0.01	18.2 ± 0.7	5.0 ± 0.4	24.6 ± 1.6	9.0 ± 0.2
6.9	11.8 ± 1.6	0.38 ± 0.04	12.0 ± 0.7	3.8 ± 0.6	-	-
8.5	10.2 ± 0.6	0.40 ± 0.03	24.0 ± 1.2	1.0 ± 0.07	26.9 ± 2.8	14.3 ± 0.6
11.2	13.2 ± 2.3	0.21 ± 0.01	20.2 ± 1.5	1.5 ± 0.3	-	-
